# Valorizing Oregano Distillation Wastewater in New Pasta Formulations: Physical, Sensory and Chemical Characteristics

**DOI:** 10.3390/foods15061092

**Published:** 2026-03-20

**Authors:** Alessandro Gallina, Maria Concetta Di Bella, Enrica Pistorio, Edoardo Marco Napoli, Maria Grazia Melilli

**Affiliations:** Institute of Biomolecular Chemistry, National Research Council ICB-CNR, Via Paolo Gaifami 18, 95126 Catania, Italy; alessandrogallina@cnr.it (A.G.); mariaconcettarita.dibella@cnr.it (M.C.D.B.); mariagrazia.melilli@cnr.it (M.G.M.)

**Keywords:** by-products, functional foods, rosmarinic acid, sustainability, wastewater

## Abstract

The agro-food industry faces significant environmental and economic challenges due to waste production. To promote sustainability, it is essential to valorize agricultural by-products, such as wastewater from essential oil distillation. Despite being a disposal burden, this wastewater retains valuable compounds with high antioxidant and antibacterial potential, making it an ideal ingredient for functional foods. Given that pasta and bread are daily staples in the Italian diet, they represent a strategic opportunity to improve public nutritional intake. In this study, a novel pasta formulation was developed by replacing 50% or 100% of the processing water with wastewater derived from oregano distillation. The investigation focused on the quality of the cooking process and the composition of phenolic compounds. A sensory analysis was also conducted to assess consumer acceptance. The enriched pasta demonstrated positive sensory characteristics, with an overall appreciation around 7, and higher levels of phenolic compounds (up to 0.79 mg GAE/g) in relation to the control sample (0.14 mgGAE/g). This highlights the potential of these rich raw materials for use in sustainable food and represents a sustainable strategy to improve the nutritional profile of pasta.

## 1. Introduction

The contemporary global context is characterized by a growing awareness of the urgency of environmental concerns, a factor that exerts a significant influence on consumer behavior, reaching its peak in 2026 thanks to an unprecedented ecological sensitivity [1]. The concept of green products is predicated on the premise that they are environmentally friendly, conserve resources, are beneficial to health, and pose no threat to the environment [2,3]. There is a broad consensus that these products are advantageous for both personal health and the environment [4,5,6,7]. Consequently, to promote sustainable development and minimize waste, it is essential to maximize the value of agricultural by-products. This is especially important considering that the agro-food industry generates considerable amounts of waste. Concomitantly, the growing focus on the link between nutrition and wellness is driving the global market towards increased demand for health-benefit products. In this context, bioactive compounds have become undisputed protagonists. They are the subject of an ever-expanding field of research, stimulating the development of new technologies and innovative products that offer added-value foods. Of particular interest are the by-products obtained from essential oil production through the extraction of aromatic plants, including spices and herbs [8].

Essential oil production processes, such as steam distillation, a common method for extracting essential oils from herbs, generate significant amounts of residual plant material and wastewater. The disposal of these by-products gives rise to significant environmental challenges, including river contamination, waste incineration, and dumping. For the food industry, this waste also represents a significant burden, requiring costly treatment and landfill. Nonetheless, since valuable volatile compounds are lost during the distillation process, the resulting waste offers an economic opportunity to recover non-volatile high-value compounds. While solid residues have received some attention from researchers [9,10,11], studies on the recovery of wastewater from essential oil extraction remain limited. Recent research has demonstrated that the effluents produced during the distillation of essential oils constitute a substantial reservoir of antioxidant phenolic compounds and organic acids. These effluents have been shown to possess antioxidant properties that are comparable to those obtained from the extraction of the essential oils themselves [12]. This essential oil wastewater represents a valuable resource for several sectors, including textiles [13], food and beverages [14], insecticides [15], and even formulations of food packaging material. The significance of this product lies in its inherent biological activities, rendering it suitable for different industrial applications, nutraceutical and pharmaceutical, including management of chronic diseases. Phenolic compounds are the primary constituents, comprising over 20/30% of dry extract [16]. In the *Lamiaceae* family, from which essential oils are obtained with yields often less than 3% of the total weight of the plant, oregano stands out for its superior effectiveness in a series of evaluations, including enzyme inhibition and antifungal protection tests, compared to many other members of the family [16]. Some research has explored the potential of wastewater and solid residues from oregano [17]. Notably, investigations conducted recently have demonstrated that wastewater from *Lamiaceae* plants, specifically oregano, rosemary and sage, possesses a remarkable capacity to impede the activities of pancreatic amylase and α-glucosidase enzymes [18,19]. These enzymes are pivotal in the process of carbohydrate degradation, leading to the formation of simple sugars.

Pasta is regularly eaten in high quantities, and it constitutes a dominant portion of the diet in many countries. Among foods functionalized with spice and herbs, pasta represents a valuable alternative, since it is a popular food widely and frequently consumed, characterized by a low glycemic index. Moreover, dried pasta is a good matrix to stabilize phytochemicals [20] that otherwise, e.g., in fresh vegetables, are easily degraded during storage, transportation and processing. The incorporation of oregano wastewater into pasta could therefore assist in the modulation of postprandial glucose absorption [18]. Furthermore, oregano distillation wastewater, naturally rich in antioxidants, is suitable to fulfill, even in part, the water needed during pasta production, without further manipulation. In this study, data concerning sensory, physical, and chemical properties of spaghetti enriched with oregano wastewater (OW) from essential oil distillation were investigated.

## 2. Materials and Methods

### 2.1. Wastewater from Oregano Distillation

Oregano (*Origanum vulgare* subsp. *hyrtum*) was kindly provided by Rinoldo Farm, located in Aragona (Agrigento, Sicily Italy), collected during the agronomic season in 2025. The hydrodistillation of the aerial parts of oregano (100 g of air-dried material) was carried out using a Clavenger-type apparatus for approximately 3 h. Following a cooling process, essential oil and hydrolates were separated and the hydrodistillation wastewater was filtered using filter paper (Whatman, cat. No. 1004-930, grade 4). The samples were stored in sealed plastic falcon tubes following their freezing until analysis.

### 2.2. Pasta Making Process

Pasta was produced with commercial durum wheat flour produced by a Sicilian mill, located in Enna (Italy). It was characterized by starch (71.2 g/100 g), protein (11.5 g/100 g) sugars (1.8 g/100 g), total fats (0.8 g/100 g), and total fiber (0.7 g/100 g). Durum wheat semolina was used as the base ingredient for all pasta formulations, with a hydration level of 28%. In the enriched samples, 50% OW and 50% salt-free tap water was used (OR50); in 100% (OR100) enriched samples, only OW was used. Pasta produced only with durum wheat semolina and salt-free tap water was used as a control (CTRL). Dough mixing was carried out for approximately 10 min using a professional pasta machine (TR50, 0.38 kW, 230 V/1/50 Hz, Catania, Italy) until a homogeneous granular consistency was obtained. The dough was then extruded through a bronze die to produce spaghetti and subsequently dried at 30 °C for 16 h in a tray dryer (Biosec, Mod. B6, Abuja, Nigeria). After drying, all pasta samples were stored at room temperature until analysis. Prior to analytical determinations, the samples were ground using a laboratory mill (IKA A11 basic) [21].

### 2.3. Determination of Pasta Color

Color analysis was performed on pasta using the Chroma meter CR-400/410 (Konica Minolta Inc., Konica Minolta, Milan, Italy). A small amount of each sample (about 10 g) was put on a watch glass. Spaghetti was cut into small pieces of about 10 cm and placed close to any empty space on the glass. Measurements were taken at three different points on all samples: this approach allowed for the accounting of the observed uneven distribution of color, across pasta surfaces. Color analysis was expressed as L*, a* and b* values, where, in a three-dimensional axes system, L* represents lightness (100 = absolute white, 0 = absolute dark), and a* (+127 = red, −127 = green) and b* (+127 = yellow, −127 = blue) are the chromaticity values. The colorimeter was calibrated using a white tile (L* = 91.48, a* = −0.18, b* = 4.50), and the analysis was performed on three repetitions.

### 2.4. Cooking Quality

The cooking quality of pasta was analyzed in terms of Optimal Cooking Time (OCT) (min), Water Absorption Index (WAI) (%), Swelling Index (SI), and Cooking Loss (CL) (%). The Optimal Cooking Time (OCT) was determined using the AACC method 66-50 [22], by examining strands of pasta at different intervals. Gentle squeezing between two glass plates allowed for observation of the disappearance of the dry, central core. Specifically, every 30 s after 4 min of cooking, a sample of pasta was taken and crushed between two plates to monitor the cooking progress. The time at which this core completely vanished was recorded as the OCT. For all other parameters, 10 g of pasta was cooked in 300 milliliters of distilled water until the OCT was reached. Afterward, the pasta and water were separated and placed in individual containers in an air oven at 105 °C for 24 h. CL was determined following this equation: (weight of drained residue in cooking water/weight of uncooked pasta) × 100 [23]. Following the method of Cleary and Brennan [24], the SI of the cooked pasta was determined (g of water per g of dry pasta). In particular, the following calculation was used: (weight of cooked product) − (weight of pasta after drying)/(weight of pasta after drying). The WAI of cooked pasta was determinate by evaluating the increase in weight of the sample following hydration during the cooking phase [25].

### 2.5. Panel Test

A sensory analysis of cooked pasta samples was conducted by a panel of ten trained panelists, comprising five women and five men, aged between 30 and 50. It was performed in the sensory test room of the CNR, in a controlled evaluation environment. The pasta from all samples was served in randomly labeled dishes for all panelists at the same time. Water was provided for rinsing purposes; to ensure the reliability of the results, panelists received specific instructions and were guided through the evaluation process, and two sessions (1 session/day; 1.5 h/session) were conducted. The panelists were selected at the CNR based on their sensory skills (ability to accurately determine and communicate the sensory attributes such as the appearance, odor, taste, and texture of a product). The panelists were also trained in sensory vocabulary and identification of attributes by evaluating durum wheat commercial spaghetti (ISO 11036, 7304).

The following sensory parameters were judged, using a 9-point hedonic scale (1 = “extremely unpleasant”, 9 = “extremely pleasant”, and 5 = “limit of acceptability”): color, appearance, odor, viscosity, elasticity, firmness, taste, and aroma. Panelists were also asked to assess the general appreciation of the sample. An explanation of the parameters has been provided for better understanding: color—visual appearance of sample, including uniformity and any discoloration; appearance—overall visual impression (including shape, size); odor—intensity and pleasantness of the aroma emanating from samples; viscosity—tendency of the paste to stick to the palate or teeth; elasticity—resistance of the pasta to deformation when bent or twisted; firmness—the amount of force that is needed to cut through the spaghetti with the front teeth.; taste—intensity and pleasantness of the flavor profile of the pasta; aroma—the intensity and pleasantness of the flavor during the consumption of the pasta; general appreciation—overall liking of samples, considering all sensory attributes. To minimize sequencing effects or bias, pasta samples were submitted anonymously and in a random order [26].

### 2.6. Chemical Characterization

#### 2.6.1. Extraction of Free Phenolic Compounds

The dried solid materials were ground to obtain powdered pasta samples. About 1 g of pasta was mixed with 3 mL of 20% *v*/*v* ethanol and kept under agitation for 16 h in the dark. Then, the mixture was centrifuged at 3500 rpm for 5 min at 4 °C and the supernatant was removed and used for the following analysis [27].

#### 2.6.2. Folin–Ciocalteu Assay

The total polyphenolic content contained in wastewater, cooked pasta and pasta cooking water was determined by the Folin–Ciocalteu method according to Abozed et al. [28] with modifications. Briefly, 0.1 mL extract solution was added to 3 mL of water and 0.5 mL of Folin–Ciocalteau reagent. The solution was mixed, and after 5 min 1.0 mL of 10% sodium carbonate was added to the mixture and put in the dark for 90 min. Absorbance was measured using a UV/VIS spectrophotometer (Agilent 8453, Cernusco sul Naviglio, Milan, Italy) at 730 nm against a blank sample. A calibration curve was built using a series of concentrations of a standard gallic acid solution, and the resulting calibration equation was used to ascertain the concentrations of phenolic acid, expressed as milligrams of equivalent gallic acid per gram of dry weight (mg GAE/g).

#### 2.6.3. Hydrodistillation Wastewater HPLC Analysis

A water solution (10 mg lyophilized wastewater/mL) of *Origanum vulgare* L. hydrodistillation wastewater was filtered on PTFE 0.45 mm filters (PALL Corporation, Port Washington, NY, USA) and put into 2 mL amber vials for analysis. Polyphenol analysis was carried out according to Sciacca et al. [18] with slight variations on an HPLC UltiMate 3000 instrument equipped with a photodiode array detector (Thermo Scientific, Rome, Italy). All chromatographic runs were performed using a reverse-phase column (Gemini C18, 250 × 4.6 mm, 5 μm, Phenomenex, Rome, Italy) equipped with a guard column (Gemini C18, 4 × 3.0 mm, 5 μm particle size, Phenomenex, Rome, Italy). Samples were eluted with a gradient of 5–90% buffer B (2.5% formic acid in acetonitrile) in buffer A (2.5% formic acid in water) over 50 min, after which the system was maintained for 7 min at 100% Buffer B. The solvent flow rate was 1 mL/min. Quantifications were carried out at 330 nm using rosmarinic acid (R^2^ = 0.998) as standard. The chemical class of each component was attributed by evaluation of their UV spectra at 330 nm, except for rosmarinic acid, identified and quantified by means of injection of the analytical standard in the same operative conditions.

#### 2.6.4. Polyphenol Characterization

All pasta samples, raw and cooked, prepared with 50% and 100% oregano hydrodistillation wastewater, were oven-dried in a cabinet dryer at 40 °C for 18 h to reduce the moisture content. After that, approximately 100 mg of each dried sample was weighed and extracted with 2 mL of a 50/50 mixture of methanol and water. The extraction was carried out for 24 h, in the dark and with continuous stirring. The resulting solution was filtered on PTFE 0.45 mm filters (PALL Corporation, Port Washington, NY, USA) and stored in vials and analyzed immediately. HPLC conditions were the same as those described in Section 2.6.3, with the same equipment and analytical protocol already used for *Salvia* wastewater.

### 2.7. Statistical Analysis

Data was submitted to Bartlett’s test for the homogeneity of variance and then analyzed using analysis of variance (ANOVA). Means were statistically separated based on Duncan’s test, when the ‘F’ test of ANOVA for treatment was significant at least at the 0.05 probability (CoHort Software Version 6.0, CoStat version 6.451). For chemical analyses, data was expressed as mean ± SD of 3 replicates of each sample. The statistical differences among pasta, OW and pasta cooking water samples were evaluated with One-Way Analysis of Variance (ANOVA) followed by All Pairwise Multiple Comparison Procedures (Holm–Sidak method). The same ANOVA analysis was conducted for sensorial attributes among cooked pasta CTRL, OR50 and OR100. Data is reported as mean ± SD of 10 replicates of each sample.

## 3. Results and Discussion

### 3.1. Colorimetric Analysis

**Figure 1:**
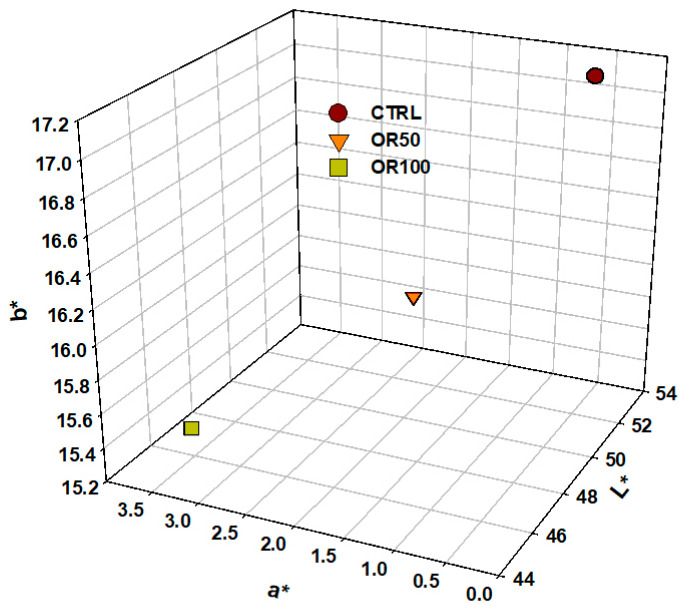
Pasta colorimetric analysis results expressed as L*, a* and b* values. LSD at *p* < 0.05 was 0.412 (L*), 0.25 (a*) and 1.00 (b*).

Color analysis revealed disparities through samples. The results expressed as mean values based on three replicas are reported in Figure 1.

**Figure 2:**
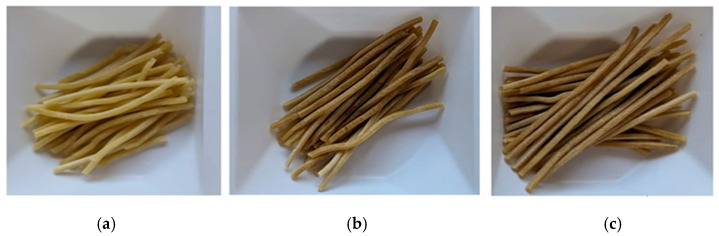
Raw pasta samples: (**a**) CTRL, (**b**) OR50 and (**c**) OR100. CTRL (control pasta); OR50 (pasta made with 50% OW and 50% salt-free tap water); OR100 (pasta made with 100% OW).

Lightness (L*) decreases progressively from CTRL (53.20) to OR100 (45.46): pasta samples are darker as more wastewater is added. A parallel tendency was noted in the b* values, whilst an opposite tendency was identified in the a* values. Pasta shows a tendency towards a slightly redder or less green hue: in fact, analysis of the red/green component (a*) reveals an increase from 1.34 to 3.39. The b* value, instead, decreases from 17.10 to 15.44. It is evident that the yellow tone undergoes a slight fading process. The OW acts as a darkening agent, introducing a slight alteration to the chroma (i.e., color intensity). This results in a shift in the overall hue of the pasta samples towards a darker, reddish color and slightly less yellow, in proportion to the wastewater concentration. The difference between colors of raw pasta samples is shown in Figure 2.

### 3.2. Cooking Quality

The cooking quality of pasta is a fundamental aspect that determines its appeal to consumers, as well as its overall sensory properties. High-quality pasta is characterized by good resistance and compactness, as well as low viscosity and limited release of organic substances into the cooking water [29]. Data on the quality of pasta is shown in Table 1.

The time at which the central starch core was no longer visible and the starch could be considered fully gelatinized, the OCT, was seven minutes in all the samples, demonstrating that substitution of water with OW does not influence the cooking time. One of the most fundamental parameters utilized to predict pasta cooking quality is Cooking Loss. The value in question indicates the quantity of dry matter lost during the cooking process into the water [30]. The analysis yielded a range of CL values, with a mean value observed that was higher in the fortified samples. Values ranged from 5.14% to 6.87%. In summary, the Cooking Loss showed by all the pasta samples was comparable and remained below the technologically acceptable limit of 8% [31]. In the analyzed samples, the increase in CL observed in fortified samples can be attributed to the dilution of gluten proteins and the consequent weakening of the starch–protein matrix. SI is indicative of the capacity of pasta to absorb water and undergo expansion during the cooking process. It is regarded as an indicator of protein integrity, which serves to prevent excessive water penetration into the pasta. Analysis of the samples showed that, while the WAI and SI both declined relative to the control at OR50, suggesting that the added material may have stabilized the pasta matrix, resulting in a more compact structure and limited water absorption during cooking, the opposite was true at OR100. The increase in WAI and SI observed in OR100 indicates that the added material weakened the protein–starch matrix at this concentration, allowing for greater and easier water penetration and resulting in a slight loss of structural strength, probably due to the electrostatic interactions between starch and carboxylic groups of rosmarinic acid [32].

### 3.3. Panel Test

The sensory evaluation results of cooked pasta are reported in Table 2. Data were analyzed by one-way ANOVA, and mean comparisons were performed using Duncan’s multiple range test (*p* < 0.05) to identify significant differences among samples. The OR100 sample exhibited the darkest color after cooking, significantly different from the other formulations, consistent with instrumental color measurements obtained for uncooked pasta.

No significant differences were observed between OR50 and OR100 samples and CTRL for viscosity and overall appreciation, as both achieved comparable mean scores.

Appearance, odor, firmness and taste were more appreciated in CTRL pasta than fortified pasta. OR50 and OR 100 were well appreciated for the elasticity sensation. Between the two fortified pastas, OR100 showed the lowest aroma score (approximately 5.33 points), but panelists appreciated the darker color. The OR50 sample displayed a high appearance score (around 7 points), despite recording the lowest color score (below 6 points), suggesting that overall visual acceptability was not solely determined by color intensity. Overall appreciation was statistically the same for the three types of pasta.

Overall, these results demonstrate that incorporation of oregano wastewater as a partial or total water replacement does not significantly compromise consumer acceptability of spaghetti compared with the control formulation.

### 3.4. Chemical Characterization

#### 3.4.1. Total Phenolic Content

The total phenolic content (TPC) of pasta and cooking water is presented in Table 3.

Significant differences were observed among the three formulations, as indicated by Duncan’s multiple range test (*p* < 0.05). The control pasta (CTRL) exhibited the lowest TPC value (0.14 mg GAE/g), whereas TPC increased substantially with the incorporation of oregano hydrodistillation wastewater (9.79 ± 0.40 mg GAE/g). Specifically, OR50 and OR100 samples showed TPC values of 0.50 and 0.79 mg GAE/g, respectively, reflecting a dose-dependent enrichment of phenolic compounds. A similar trend was observed in the cooking water. While the CTRL sample released minimal phenolics into the water (0.11 mg GAE/g), OR50 and OR100 cooking waters contained significantly higher levels (0.32 and 0.57 mg GAE/g, respectively), indicating leaching of bioactive compounds during the cooking process. These results demonstrate that replacement of water with oregano wastewater effectively increases the phenolic content of the pasta matrix. The observed increase in TPC aligns with previous studies on functional pasta enrichment, suggesting that phenolic compounds from herbal by-products can be successfully incorporated into pasta without negatively affecting technological properties [33]. Moreover, the dose-dependent response between OR50 and OR100 provides useful guidance for optimizing enrichment levels to balance bioactivity with sensory acceptability, as discussed in the panel test results.

#### 3.4.2. HPLC Determination of Polyphenol Content of Wastewater and Pasta Samples

Polyphenols quantified for all samples are reported in Table 4.

HPLC analysis of oregano hydrodistillation wastewater identified one cinnamic acid, rosmarinic acid, and four flavonoids as the main bioactive components. The total polyphenol content, expressed as rosmarinic acid equivalents, was 0.34 mg/mL, in agreement with previous reports [28], confirming the high biological value of this by-product. Among the phenolics, rosmarinic acid was the predominant compound, reaching 0.25 mg/mL.

It is reported in the literature that cinnamic acid derivatives have antioxidant and antimicrobial properties [34], while rosmarinic acid has been reported to possess several beneficial health properties such as hepatoprotective, anti-inflammatory and neuroprotective activity [35], so the addition of OW to pasta formulations can contribute to enhancing the added value of this food.

Analysis of the control (CTRL) pasta revealed that none of these bioactive compounds were detectable.

Raw pasta prepared with 100% hydrodistillation wastewater exhibited a total polyphenol content of 88.04 mg/100 g d.w., of which 75.39 mg/100 g d.w. was rosmarinic acid. After cooking, these values decreased to 40.91 mg/100 g d.w. and 31.50 mg/100 g d.w., respectively, yet remained substantially high, demonstrating the dough’s strong retention capacity during thermal processing. Pasta prepared with 50% wastewater (OR50) also showed an elevated polyphenol content, with slightly lower absolute values, but the retention after cooking remained effective. This is further supported by analysis of the cooking water: bioactive metabolites released from OR50 pasta were approximately half of those released from OR100, consistent with the reduced proportion of wastewater in the dough.

**Figure 3:**
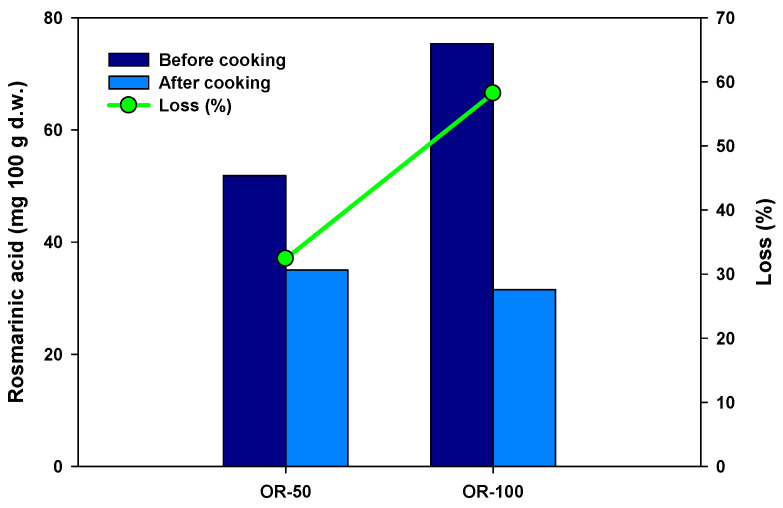
Rosmarinic acid content in fortified pasta samples before and after cooking. OR50 (pasta made with 50% OW and 50% salt-free tap water); OR100 (pasta made with 100% OW).

These trends are illustrated in Figure 3. Despite the lower initial rosmarinic acid content in OR50 pasta (51.86 vs. 75.39 mg/100 g d.w. in OR100), the compound was efficiently retained during cooking. The percentage loss of rosmarinic acid was 32.4% for OR50 and 58.2% for OR100. These findings highlight the strong retention of polyphenols during pasta processing, even when the dough is enriched with high levels of bioactive compounds.

## 4. Conclusions

The results highlight the potential of oregano distillation wastewater as a sustainable and functional food ingredient. Its use allows the production of pasta with enhanced nutritional value, providing a practical strategy to deliver bioactive polyphenols in the diet while valorizing agro-industrial by-products and supporting environmentally friendly practices.

OW fortified pasta substantially increased the total polyphenol content vs. CTRL; despite OR 100 exhibiting a higher amount of rosmarinic acid, the cooking process led to a loss of 58.2% *vs.*. 32.4% recorded in OR50, demonstrating the dough’s strong retention capacity. Moreover, for OR50 pasta, the enrichment did not negatively affect technological properties or sensory attributes; in particular, cooking quality assessment showed the lowest WAI and SI results compared to the control sample. The panel test showed a comparable or even slightly improved acceptability (higher elasticity and aroma scores than the control); the colorimetric analysis, instead, showed a darker color and higher red and blue hues as more OW was added to pasta. Furthermore, OR100 showed a high value for Cooking Loss, while for sensory attributes, there was a significative increase in color, due to the higher amount of OW added that led to a rise in the “overall appreciation” score, which in any case was not statically different from OR50.

On the basis of the obtained results, the pasta fortified with 50% OW showed a good compromise among the uptake of chemical bioactive compounds, sensory characteristics, and good cooking quality parameters.

While further large-scale studies are needed, the recovery and use of oregano distillation wastewater represent a sustainable strategy to improve the nutritional profile of pasta, meet the growing demand for “green” products, and reduce the environmental impact of agro-industrial by-products.

## Figures and Tables

**Table 1:** Cooking quality measurements results.

Pasta Sample	OCT (min:ss)	WAI (%)	CL (%)	SI (g Water per g Dry Pasta)
CTRL	7:00	181.75 ± 0.94 ^a^	5.14 ± 0.06 ^c^	3.41 ± 0.18 ^a^
OR50	7:00	174.50 ± 4.70 ^b^	5.82 ± 0.39 ^b^	3.33 ± 0.02 ^a^
OR100	7:00	176.22 ± 2.35 ^ab^	6.87 ± 0.15 ^a^	3.41 ± 0.07 ^a^

Superscript letters in each column indicate differences at *p* < 0.05 among samples. CTRL (control pasta); OR50 (pasta made with 50% OW and 50% salt-free tap water); OR100 (pasta made with 100% OW).

**Table 2:** Sensory attributes of the samples.

Attribute	CTRL	OR50	OR100
Color	6.17 ± 1.47 ^b^	5.67 ± 0.82 ^c^	7.00 ± 0.00 ^a^
Appearance	7.33 ± 1.75 ^a^	6.83 ± 1.33 ^b^	6.67 ± 0.82 ^b^
Odor	7.67 ± 1.75 ^a^	7.00 ± 1.10 ^b^	7.00 ± 1.10 ^b^
Viscosity	6.17 ± 0.41 ^a^	6.17 ± 0.75 ^a^	6.00 ± 0.63 ^a^
Elasticity	5.83 ± 0.41 ^b^	6.33 ± 1.21 ^a^	6.50 ± 1.05 ^a^
Firmness	6.50 ± 1.38 ^a^	6.17 ± 1.17 ^ab^	6.00 ± 0.89 ^b^
Taste	6.83 ± 1.17 ^a^	6.17 ± 0.75 ^b^	5.83 ± 1.17 ^b^
Aroma	5.67 ± 1.97 ^a^	5.83 ± 2.56 ^a^	5.33 ± 2.16 ^b^
Overall appreciation	7.00 ± 0.63 ^a^	6.67 ± 0.52 ^a^	7.17 ± 0.98 ^a^

Superscript letters in each row indicate differences at *p* < 0.05 among samples. CTRL (control pasta); OR50 (pasta made with 50% OW and 50% salt-free tap water); OR100 (pasta made with 100% OW).

**Table 3:** Total phenolic content (mg GAE/g) of the studied samples.

	Pasta Sample	Cooking Water
CTRL	0.14 ± 0.02 ^c^	0.11 ± 0.00 ^c^
OR50	0.50 ± 0.02 ^b^	0.32 ± 0.04 ^b^
OR100	0.79 ± 0.03 ^a^	0.57 ± 0.04 ^a^

Superscript letters in each column indicate differences at *p* < 0.05 among samples. CTRL (control pasta); OR50 (pasta made with 50% OW and 50% salt-free tap water); OR100 (pasta made with 100% OW).

**Table 4:** Total phenolic characterization in all samples. Results are means of 3 replicates ± SD.

Compound	OW	OR50 Before Cooking	OR50 After Cooking	OR50 Residual Water	OR100 Before Cooking	OR100 After Cooking	OR100 Residual Water
	(mg/mL)	(mg/100 g d.w.)					
Cinnamic acid	0.02 ± 0.00	3.54 ± 0.11 ^b^	2.58 ±0.02 ^d^	0.92 ± 0.01 ^f^	4.16 ± 0.00 ^a^	2.81 ± 0.01 ^c^	1.86 ± 0.05 ^e^
Rosmarinic acid	0.25 ± 0.00	51.86 ± 1.36 ^b^	35.04 ± 0.32 ^c^	7.42 ± 0.33 ^f^	75.39 ± 0.25 ^a^	31.50 ± 1.90 ^d^	18.44 ±0.54 ^e^
Flavonoid 1	0.01 ± 0.00	3.76 ± 0.09 ^a^	2.43 ± 0.02 ^b^	0.92 ± 0.00 ^c^	4.27 ± 0.05 ^a^	2.19 ± 0.64 ^b^	1.79 ±0.04 ^b^
Flavonoid 2	0.03 ± 0.00	2.71 ± 0.08 ^b^	1.72 ± 0.03 ^c^	0.68 ± 0.02 ^b^	3.21 ± 0.09 ^a^	1.45 ± 0.44 ^c^	1.42 ±0.03 ^c^
Flavonoid 3	0.01 ± 0.00	5.48 ± 0.76 ^b^	3.79 ± 0.09 ^c^	1.24 ± 0.00 ^d^	8.03 ± 0.20 ^a^	2.74 ± 0.80 ^c^	2.71 ±0.04 ^c^
Flavonoid 4	0.01 ± 0.00	1.48 ± 0.04 ^b^	1.34 ± 0.02 ^b^	0.47 ± 0.01 ^e^	2.60 ± 0.10 ^a^	0.72 ± 0.22 ^d^	1.02 ±0.01 ^c^
Total Phenols	0.34 ± 0.00	68.83 ± 2.43 ^b^	46.90 ± 0.50 ^c^	11.67 ± 0.38 ^e^	88.04 ± 13.13 ^a^	40.91 ± 4.70 ^cd^	27.25 ±0.69 ^d^

Superscript letters in each row indicate significant differences (*p* < 0.05). OW was not included in the statistical analyses. OR50 (pasta made with 50% OW and 50% salt-free tap water); OR100 (pasta made with 100% OW).

## Data Availability

The original contributions presented in the study are included in the article. Further inquiries can be directed to the corresponding authors.
